# Human Leucocyte Antigen Genetics in Idiosyncratic Drug-Induced Liver Injury with Evidence Based on the Roussel Uclaf Causality Assessment Method

**DOI:** 10.3390/medicines11040009

**Published:** 2024-04-11

**Authors:** Rolf Teschke, Gaby Danan

**Affiliations:** 1 Department of Internal Medicine II, Division of Gastroenterology and Hepatology, Klinikum Hanau, D-63450 Hanau, Germany; 2 Academic Teaching Hospital of the Medical Faculty, Goethe University Frankfurt/Main, D-60590 Frankfurt am Main, Germany; 3 Pharmacovigilance Consultancy, Rue Des Ormeaux, 75020 Paris, France; gaby.danan@gmail.com

**Keywords:** Roussel Uclaf causality assessment method, updated RUCAM, DILI, idiosyncratic drug-induced liver injury, human leucocyte antigen, iDILI genetics

## Abstract

The human leucocyte antigen (HLA) allele variability was studied in cohorts of patients with idiosyncratic drug-induced liver injury (iDILI). Some reports showed an association between HLA genetics and iDILI, proposing HLA alleles as a potential risk factor for the liver injury. However, the strength of such assumptions heavily depends on the quality of the iDILI diagnosis, calling for a thorough analysis. Using the PubMed database and Google Science, a total of 25 reports of case series or single cases were retrieved using the terms HLA genes and iDILI. It turned out that in 10/25 reports (40%), HLA genetics were determined in iDILI cases, for which no causality assessment method (CAM) was used or a non-validated tool was applied, meaning the findings were based on subjective opinion, providing disputable results and hence not scoring individual key elements. By contrast, in most iDILI reports (60%), the Roussel Uclaf Causality Assessment Method (RUCAM) was applied, which is the diagnostic algorithm preferred worldwide to assess causality in iDILI cases and represents a quantitative, objective tool that has been well validated by both internal and external DILI experts. The RUCAM provided evidence-based results concerning liver injury by 1 drug class (antituberculotics + antiretrovirals) and 19 different drugs, comprising 900 iDILI cases. Among the top-ranking drugs were amoxicillin–clavulanate (290 cases, HLA A*02:01 or HLA A*30:02), followed by flucloxacillin (255 cases, HLA B*57:01), trimethoprim–sulfamethoxazole (86 cases, HLA B*14:01 or HLA B*14:02), methimazole (40 cases, HLA C*03:02), carbamazepine (29 cases, HLA A*31:01), and nitrofurantoin (26 cases, HLA A*33:01). In conclusion, the HLA genetics in 900 idiosyncratic drug-induced liver injury cases with evidence based on the RUCAM are available for studying the mechanistic steps leading to the injury, including metabolic factors through cytochrome P450 isoforms and processes that activate the innate immune system to the adaptive immune system.

## 1. Introduction

Prescribed drugs are commonly well tolerated, but some medicines may cause idiosyncratic drug-induced liver injury (iDILI) in a few individuals [1,2,3,4,5]. The rarity and unpredictable occurrence of iDILI in the general population represent a clinical challenge for studying its clinical features [6,7], but options are available for this human disease to be used as an excellent human study model of iDILI, provided the clinical diagnosis is correct [8]. Often found in published reports on cases of iDILI, alternative causes as confounding variables may invalidate the iDILI diagnosis [9], and the diagnosis of iDILI cases provided by the US LiverTox database remains debated due to not being properly assessed for causality [10,11,12]. Similarly, the characteristics of iDILI are often based on cases presented as narratives or assessed by the US DILIN method [3], which lacks proper validation via cases with a positive re-exposure test and is based on arbitrary, subjective opinions and confined to the US territory. The diagnostic problems of iDILI are best circumvented by applying the original Roussel Uclaf Causality Assessment Method (RUCAM) from 1993 [13,14], or better still, the updated RUCAM version from 2016 [15] in line with recommendations by other groups [1,5,6,16,17,18,19,20,21,22,23,24,25,26,27,28,29,30,31,32,33,34,35,36,37,38,39,40,41], including the Chinese Drug-Induced Liver Injury (DILI) Study Group of the Chinese Society of Hepatology (CSH) and Chinese Medical Association (CMA), as published in their CSH guidelines for the diagnosis and treatment of drug-induced liver injury [42]. 

Because this form of DILI is idiosyncratic, it is highly suggestive of the genetic susceptibility of the exposed individual [3,6]. Much of the efforts focused on the role of the human leucocyte antigen (HLA) alleles associated with iDILI are based on specific drugs or ethnicities [6]. The quality of reports on HLA’s association with iDILI is variable: many reports used the RUCAM for the causality assessment, some reports included final RUCAM-based causality rankings [43,44,45,46,47,48,49,50,51,52,53,54,55,56,57,58,59], but other reports used no causality assessment method (CAM) at all or a non-validated method based on vague opinion only [60,61,62,63,64,65,66,67,68,69,70].

This analysis focused on patients with iDILI, the HLA association, and the established diagnosis as evidenced by RUCAM use. Other studies with diagnostic shortcomings were also discussed with proposals to improve the quality of future case reports on the HLA association.

## 2. Search Terms and Strategy

The literature search strategy involved the PubMed database (https://pubmed.ncbi.nlm.nih.gov/ accessed 15 December 2023) and Google Scholar (https://scholar.google.com accessed 15 December 2023 and using the following terms were used: idiosyncratic drug-induced liver injury, RUCAM, updated RUCAM, HLA, and combinations thereof. The first 50 publications derived using each term group were checked for their suitability to be included in this review article and provided the primary basis for further analysis. The search was performed first on 30 December 2023 and then finalized on 25 January 2024. Some additional reports may have been excluded from the analysis due to not consulting other sources apart from Google Scholar. The search was limited to publications in the English language, but there were no other restrictions regarding the year of publication or study design. 

## 3. Drugs Causing RUCAM-Based iDILI Cases with HLA Association 

The HLA genetics were verified for 19 drugs and 1 drug class in a total of 900 cases of iDILI with evidence based on the RUCAM for the causality assessment, as reported in 16 publications (Table 1) [43,44,45,46,47,48,49,50,51,52,53,54,55,56,57,58,59]. In 683/900 iDILI cases (76%), the RUCAM-based final scores or causality gradings were presented, ranging from possible to highly probable causality gradings in most study cohorts. The inclusion of cases with a possible causality ranking is problematic, as this confounds the valid results obtained from cases with a probable or highly probable causality level. Possible causality levels are commonly due to a retrospective study protocol with incomplete data collection and to neglecting alternative causes, thus calling for prospective studies as the best analytical approach [15]. 

At the top of the drugs most commonly implicated in RUCAM-based iDILI with HLA analysis was amoxicillin–clavulanate, followed by flucloxacillin, trimethoprim–sulfamethoxazole, methimazole, carbamazepine, and nitrofurantoin, with case numbers ranging from 1 to 201 (Table 1). 

## 4. Drugs Causing iDILI Cases with Unverified Diagnosis and Suspected HLA Association

Highly problematic were the studies on HLA alleles in cases of iDILI not assessed for causality by the RUCAM or assessed by the disputed as not validated Drug-Induced Liver Injury Network (DILIN) method based on arbitrary, subjective opinion (Table 2) [60,61,62,63,64,65,66,67,68,69,70].

## 5. Characteristics of the RUCAM versus the DILIN Method 

The global view on the causality assessment of iDILI cases shows that the RUCAM outperforms any other tool in terms of case numbers [3], with 81,856 DILI cases assessed by the RUCAM and published worldwide from 1993 to mid-2020 [4]. Summarized are the advantages of the RUCAM’s specifics (Table 3) [3].

RUCAM use requires both an assessor familiar with the issues of DILI and complete case data to obtain the best causality gradings, as the RUCAM cannot compensate for missing prerequisites, leading to challenges and limitations of the RUCAM (Table 4) [3]. 

As opposed to many other tools, the RUCAM received both internal and external validation by experts in the field (Table 5) [3].

The US network method is based on non-defined and non-scored elements as well as on arbitrary subjective opinion, which does not allow for a robust validation. The results of HLA studies remain a matter of debate (Table 2). Together with many other methodological shortcomings, the results of this tool are disappointing due to the known issues published in detail (Table 6) [81].

## 6. Drugs, iDILI, and Lack of HLA Association 

No significant HLA association was detected for some drugs and drug classes implicated in causing iDILI (Table 7). 

## 7. HLA Genetic Association with RUCAM-Based iDILI by Some Drugs

HLA gene analysis of iDILI cases with a firm diagnosis as evidence based on the RUCAM indicates a genetic association with the injury caused by 19 drugs and 1 drug class, but there is variability in the HLA allele genes responsible for individual drugs triggering the liver injury (Table 1) [43,44,45,46,47,48,49,50,51,52,53,54,55,56,57,58,59]. This association could not be firmly established in iDILI cases lacking a robust causality assessment (Table 2) [60,61,62,63,64,65,66,67,68,69,70]. Finally, several drugs caused iDILI by a non-HLA process (Table 7). 

## 8. Molecular Considerations of the Liver Injury 

The mechanistic steps in iDILI evidenced by the RUCAM and association with HLA were discussed in some liver injury cases for a limited number of drugs (Table 1) [43,44,45,46,47,48,49,50,51,52,53,54,55,56,57,58].

### 8.1. Amoxicillin and Amoxicillin–Clavulanate

For the 15 RUCAM-based iDILI cases and HLA association, no molecular details were published on how amoxicillin causes the liver injury [43]. In addition, studies on the HLA genotypes on the susceptibility to amoxicillin–clavulanate in 201 RUCAM-based iDILI cases confirmed the iDILI caused by amoxicillin–clavulanate as an immune disease, whereby the gene product may have a general role in the regulation of T-cell responses, and suggested the importance of the adaptive immune response in the pathogenesis, but details of the molecular involvement were not provided [44]. There was speculation in another 75 RUCAM-based cases of iDILI caused by amoxicillin–clavulanate that the underlying molecular mechanism of the liver injury could involve protein–drug/metabolite complex presentation by HLA class I and II molecules, followed by T-cell mediated cytotoxicity and cytokine generation [45]. To expand the mechanistic speculation, the expression of HLA class I molecules on hepatocytes could contribute to hepatocellular injury, whereas HLA class II antigens have been detected on biliary epithelium cells, which may trigger the cholestatic type of injury. There is a concomitant note on the role of natural killer cells with their abundant presence in the liver, whose level of cytotoxic responsiveness is largely influenced by HLA I class binding [45]. In the third HLA study, with 14 RUCAM-based iDILI cases due to amoxicillin–clavulanate, overexpression of the HLA class II molecules on the biliary epithelial cells was considered as a possible initiating process for the autoimmune-mediated bile duct destruction metabolically associated with the formation of neoantigens and their recognition as foreign by the immune system [46]. This immune mechanism is supported by the requirement of HLA class II molecules for the antigen presentation to CD4 positive T cells.

### 8.2. Antituberculotics + Antiretrovirals

In HLA studies on 46 RUCAM-based iDILI due to concomitant use of antituberculotic and antiretroviral drugs, no possible mechanistic steps for the liver injury were proposed, likely due to the fact that two different drug groups were used rather than single drugs [47]. 

### 8.3. Carbamazepine

For HLA studies on 29 cases of RUCAM-based DILI due to carbamazepine, no mechanistic proposals were provided [48]. 

### 8.4. Dapsone

In four RUCAM-based cases of iDILI due to dapsone with HLA association, a reminder was published that HLA association does not prove causation, because studies are required directly showing the presence of cytotoxic T lymphocytes in the liver [49]. As a cautious proposal in view of the low case number, exposure to dapsone may promote the activation and recruitment of toxic T lymphocytes from the circulation to the liver, and the release of cytokines like granulysin, tumor necrosis factor-alpha, and interferon-gamma may initiate the liver injury. 

### 8.5. Enalapril

As expected in the face of the low case number, no mechanistic proposals have been presented for the four RUCAM-based iDILI cases caused by enalapril with HLA association [50]. 

### 8.6. Erythromycin

For 10 RUCAM-based cases of iDILI caused by erythromycin with established HLA association, no suggestions of possible mechanistic steps leading to the liver injury were presented [50]. 

### 8.7. Fenofibrate

There is a lack of molecular details in seven RUCAM-based iDILI due to fenofibrate in association with HLA [50].

### 8.8. Flucloxacillin

With 255 RUCAM-based iDILI cases caused by flucloxacillin and the HLA B*57:01 genotype, this is the second largest and best described iDILI cohort by a single drug (Table 1) [43,51,52,53]. Molecular aspects were not discussed in the HLA study on 51 RUCAM-based cases of iDILI due to flucloxacillin [51]. However, molecular details were presented in the HLA study comprising six RUCAM-based cases of iDILI caused by flucloxacillin, supporting the role of the adaptive immune system [52]. More specifically, studies using flucloxacillin-responsive CD41 and CD81 T cells from patients with liver injury showed that naive CD45RA1CD81 T cells from volunteers expressing HLA-B*57:01 were activated with flucloxacillin when dendritic cells present the drug antigen. T cell clones expressing CCR4 and CCR9 migrated toward CCL17 and CCL 25, and they secreted interferon-gamma (IFN-c), T helper (Th)2 cytokines, perforin, granzyme B, and FasL following drug stimulation. Flucloxacillin bound covalently to selective lysine residues on albumin in a time-dependent manner and the level of binding correlated directly with the stimulation of the clones. Activation of CD81 clones with flucloxacillin was process-dependent and restricted by HLA-B*57:01 and the closely related HLA-B*58:01 [52]. In another HLA study comprising 197 RUCAM-based cases with iDILI caused by flucloxacillin, previous proposals were reiterated without the addition of new ones [43]. No new mechanistic ideas were presented in an HLA study with one RUCAM-based case of iDILI due to flucloxacillin [53]. 

### 8.9. Flupirtine 

Within the HLA study comprising 11 RUCAM-based cases of iDILI caused by flupirtine, the proposal was made that the liver injury likely involves an inappropriate T cell response within the liver, supporting the concept that the adaptive immune system is involved in the pathogenesis of this iDILI [54]. 

### 8.10. Infliximab 

In the context of 18 RUCAM-based cases of iDILI caused by infliximab with HLA association, possible mechanisms by which the biologic drug interacts with the HLA allele B*39:01 are unknown and wait for future studies [55]. 

### 8.11. Isoxazolyl Penicillins

This drug group includes cases of dicloxacillin (*n* = 2), cloxacillin (*n* = 2), and oxacillin (*n* = 2), but not those of flucloxacillin [43]. In the HLA study of RUCAM based on these six cases of iDILI, no specific mechanistic aspects were presented.

### 8.12. Methimazole 

In the HLA study on 40 RUCAM-based cases of iDILI due to methimazole, docking studies revealed that methimazole could bind indirectly to HLA [56]. The hapten hypothesis may explain the mechanism of HLA involvement in iDILI by methimazole, where methimazole or its metabolites are supposed to covalently bind to cellular proteins, leading to the production of drug–peptide adducts to T cells via HLAs. 

### 8.13. Methyldopa 

For the HLA study comprising 4 RUCAM-based cases of iDILI due to methyldopa, no mechanistic proposals were made [50]. 

### 8.14. Minocycline 

Mechanistic proposals derived from 25 RUCAM-based cases of iDILI caused by minocycline in association with HLA include the direct molecular docking of minocycline to the HLA allele HLA B*35:02 as an important initiating step in iDILI by minocycline in support of a role for adaptive immunity [57]. 

### 8.15. Nitrofurantoin 

Mechanistic proposals were not presented following analysis of 26 RUCAM-based cases of iDILI caused by nitrofurantoin [58]. 

### 8.16. Sertaline

HLA studies on five RUCAM-based cases of iDILI caused by sertaline revealed no clues as to the molecular steps leading to the liver injury [50]. 

### 8.17. Terbinafine 

Considering 14 RUCAM-based cases of iDILI caused by terbinafine with HLA association, mechanistic proposals primarily focused on metabolic aspects, since *N*-dealkylation leads to the generation of an aldehyde metabolite that reacts with glutathione [50]. This GSH-adduct is transported across the canalicular membrane and concentrated in the bile, where it may injure the biliary epithelial cells. Most interesting, treatment of monocytes with terbinafine causes the release of the proinflammatory cytokines IL-8 and TNF-alpha. However, there was no evidence of a role of CYP genes or innate immune genes in the liver injury cases, although several CYP isoforms are involved in the metabolism of the parent drug [50]. 

### 8.18. Ticlopidine 

HLA studies on five RUCAM-based cases of iDILI due to ticlopidine failed to provide the mechanistic and molecular aspects leading to the liver injury [50]. 

### 8.19. Trimethoprim–Sulfamethoxazole

HLA studies on 86 RUCAM-based cases of iDILI caused by trimethoprim–sulfamethoxazole showed molecular docking in HLA to be the predictive sites for the drug metabolites [59]. 

## 9. Specific Molecular Aspects of HLA in iDILI 

HLA is located on the human chromosome six short arm and represents a complex consisting of several tightly linked genes, which encode the major histocompatibility complex (MHC) to regulate immunity [83]. Drugs implicated in iDILI are mostly metabolized by hepatic microsomal CYP isoforms [84,85] or rarely by other hepatic enzymes, including aldehyde oxidase, aldehyde dehydrogenase 1A1 and 3A1, alcohol dehydrogenase 1A, 1C, and 4, flavin-containing monooxygenases 2, 3, and 5, and xanthine oxidase, which can also produce toxic metabolites [86,87,88]. The drug or its reactive metabolites function as haptens, bind to proteins, and then form neoantigens that present on specific HLA molecules with the risk of triggering an inappropriate immune response that contributes to the liver injury [83,89]. 

Consensus exists that the association of specific HLA genotypes with iDILI caused by some drugs provides strong evidence that it is mediated by the adaptive immune system [83,85,89,90]. This is also consistent with the liver histology of iDILI, showing a monocytic inflammatory infiltrate as a typical immune reaction and the specific clinical immune characteristics of iDILI like skin rash [90]. The initiation of an immune response requires the activation of antigen-presenting cells (APCs) by molecules such as danger-associated molecular pattern molecules (DAMPs) [83,85,89,90]. An attractive hypothesis for the mechanism by which DAMPs induce an immune response is through the activation of inflammasomes [83,89,90]. The dominant immune response in the liver is immune tolerance, and it is only when immune tolerance fails that significant liver injury occurs [83,89]. Although it appears that the liver damage is mediated by the adaptive immune system, an innate immune response is required for an adaptive immune response [89], an opinion supported by a study on HLA associated with RUCAM-based iDILI caused by flucloxacillin, whereby human leukocyte antigen (HLA)-B*57:01-restricted activation of drug-specific T cells providing the immunological basis for flucloxacillin-induced liver injury [52]. However, there is room for non-HLA mechanisms in iDILI, as shown so far for a few drugs (Table 7) [82], especially in view of the fact that most drugs implicated in 81,856 RUCAM-based iDILI cases were not yet evaluated for HLA association [4]. Considering the complexity of the HLA association with iDILI, a few key facts on mechanistic aspects of the role of HLA in iDILI are provided as a listing (Table 8).

## 10. Proposals for Future Studies

For future cases, the following points should be considered: (1) the HLA gene association with specific drugs causing iDILI should be studied with iDILI cases assessed prospectively for causality by the updated RUCAM [15,90]; (2) only cases with a probable or highly probable RUCAM-based causality grading should be included in the study cohort, meeting the classical thresholds of alanine aminotransferase (ALT) ≥ 5 times the upper limit of normal (ULN) and/or alkaline phosphatase (ALP) ≥ 2 times the ULN, provided it is of hepatic origin [15]; (3) a prospective study protocol is preferred to achieve complete case datasets and a chance to reach high causality gradings [15], although the updated RUCAM can also handle data from retrospective studies with the risk of lower causality gradings [15]; (4) the use of causality assessment tools based on arbitrary opinion and not validated should be discouraged (Table 6) and refused for publication to reduce confusion and background noise; and finally, (5) new study approaches are required in RUCAM-based iDILI cases quantifying circulatory mediators derived from the injured that may mirror what happens within the liver regarding the activation of the innate immune system to the adaptive immune system [83,89,90]. 

## 11. Conclusions

There is strong RUCAM-based evidence that HLA gene variability is associated with iDILI due to selected drugs, but a mere association does not necessarily mean causation. It is also obvious that studies, which fail to use a robust, validated, and quantitative causality assessment method, will be unable to provide evidence-based data and cannot contribute to the knowledge of the mechanistic steps involved in iDILI. Yet, a major gap exists between HLA genes and the emerging iDILI as established diagnosis. Around this gap, much is speculated on the possible intermediates, role of CYP isoforms, reactive oxygen species, hapten mechanism through covalent binding with formation of drug–protein conjugates, and the activation process transforming the innate immune system into the adaptive immune system that may finally trigger the idiosyncratic liver injury. Under these conditions, a strong human study model as a clinical cohort is required, including the updated RUCAM to obtain reliable iDILI cases and to establish a firm association with HLA groups that would add to the knowledge of the steps leading to the liver injury. 

## Figures and Tables

**Table 1:** Drugs causing iDILI assessed for HLA association and causality by the RUCAM.

Drug	HLA Allele	RUCAM-Based iDILI Cases (*n*)	RUCAM-Based Causality	First Author
Amoxicillin	A*01:01 C*03:02 B*58:01 DPB1*01:01	15	Not specified	Nicoletti, 2019 [43]
Amoxicillin–Clavulanate	A*02:01 DQB1*06:02	201	A total of 14/201 patients had a possible causality, and 187 a probable or highly probable causality grading	Lucena, 2011 [44]
Amoxicillin– Clavulanate	A*30:02 B*18:01 DRB1*15:01 DQB1*06:02	75	Possible causality and higher	Stephens, 2013 [45]
Amoxicillin–Clavulanate	DRB1*15:01	14	Not specified	O’Donohue, 2000 [46]
Antituberculotics + Antiretrovirals	B*57:02 B*57:03	46	A total of 4/46 patients had a possible causality grading, 12 a probable, and 30 a highly probable causality	Petros, 2017 [47]
Carbamazepine	A*31:01	29	All patients had a possible causality and higher	Nicoletti, 2019 [48]
Dapsone	B*13:01	4	Highly probable causality	Devarbhavi, 2022 [49]
Enalapril	A*33:01	4	Not specified	Nicoletti, 2017 [50]
Erythromycin	A*33:01	10	Not specified	Nicoletti, 2017 [50]
Fenofibrate	A*33:01	7	Not specified	Nicoletti, 2017 [50]
Flucloxacillin	B*5701	51	A total of 4/51 patients had a possible causality, 18 a probable causality, and 29 a highly probable causality grading	Daly, 2009 [51]
Flucloxacillin	B*57:01	6	A total of 2/6 patients had a possible causality, 2 a probable, and 2 a highly probable causality	Monshi, 2013 [52]
Flucloxacillin	B*57:01	197	A total of 22/197 patients had a possible causality, 90 a probable, and 85 a highly probable causality grading	Nicoletti, 2019 [43]
Flucloxacillin	B*57:01	1	Score 8, probable causality	Teixera, 2020 [53]
Flupirtine	DRB1*16:01-DQB*05:02	11	A total of 1/11 patients had an unlikely causality grading, 5 a possible, and 5 a probable causality grading	Nicoletti, 2016 [54]
Infliximab	B*39:01	18	Not specified	Bruno, 2020 [55]
Isoxazolyl Penicillins	C*07:04 DQB1*06:09	6	Not specified	Nicoletti, 2019 [43]
Methimazole	C*03:02	40	A total of 1/40 patients had a possible causality grading, 37 a probable, and 2 a highly probable causality grading	Li, 2019 [56]
Methyldopa	A*33:01	4	Not specified	Nicoletti, 2017 [50]
Minocycline	B*35:02	25	Not specified	Urban, 2017 [57]
Nitrofurantoin	A*33:01 DQB1*02:02 A*30:02 DQA1*02:01 DRB1*07:01 DPB1*16:01 C*06:02	26	A total of 18/26 patients had a score of above 6, in line with a probable or highly probable causality	Daly, 2023 [58]
Sertaline	A*33:01	5	Not specified	Nicoletti, 2017 [50]
Terbinafine	A*33:01	14	Not specified	Nicoletti, 2017 [50]
Ticlopidine	A*33:01	5	Not specified	Nicoletti, 2017 [50]
Trimethoprim–Sulfamethoxazole	B*14:01 B*14:02 B*35:01	86	Not specified	Li, 2021 [59]

As in most studies patients provided written informed consent and the study was approved by the institutional board, the studies likely initially followed a prospective study protocol with retrospective analysis of the obtained data [43,53,54,55,56,57,58,59]. Abbreviations: iDILI, idiosyncratic drug induced liver injury; HLA, human leucocyte antigen; RUCAM, Roussel Uclaf Causality Assessment Method.

**Table 2:** Drugs causing iDILI evaluated for underlying HLA association but not assessed by the RUCAM.

Drug	HLA Allele	iDILI Cases (n)	Causality Assessment Method	First Author
Allopurinol	A*34:02 B*53:01 B*58:01	11	No RUCAM but DILIN method	Fontana, 2021 [60]
Allopurinol	B*58:01	3	None	Kim, 2017 [61]
Amoxicillin– Clavulanate	DRB1*1501 DQB1*0602	35	None	Hautekeete, 1999 [62]
Halothane	*DR2*	14	None	Otsuka, 1985 [63]
Lapatinib	DRB1*07:01	65	None	Tangamornsuksan, 2020 [64]
Lumiracoxib	DRB1*15:01	139	None	Singer, 2010 [65]
Nitrofurantoin	DRB1*11:04	78	No RUCAM but DILIN method	Chalasani, 2023 [66]
Pazopanib	B*57:01 C*04:01 C*06:02	2190	None	Xu, 2016 [67]
Terbinafine	A*33:01	15	No RUCAM but DILIN method	Fontana, 2018 [68]
Ticlopidine	A*33:03	22	None	Hirata, 2008 [69]
Ximelagatran	DRB1*07 DQA1*02	74	None	Kindmark, 2008 [70]

Some cases were characterized by severe cutaneous adverse reactions (SCARs) like Stevens–Johnson syndrome (SJS), toxic epidermal necrolysis (TEN), and drug reaction with eosinophilia and systemic symptoms (DRESSs) [61]. Abbreviations: DILIN, drug-induced liver injury network; iDILI, idiosyncratic drug-induced liver injury; HLA; human leucocyte antigen; RUCAM, Roussel Uclaf Causality Assessment Method.

**Table 3:** Advantages of the RUCAM.

RUCAM with Its Basic Features and Specifics
● Fully validated method based on cases with positive re-exposure test results (gold standard), thereby providing a robust CAM [5,14]
● External validation by inter-rater reliability in 3 studies [71,72,73]
● Worldwide use, with 81,856 DILI cases assessed by the RUCAM published up to mid-2020, thereby outperforming any other CAM in terms of the number of cases published [4]
● Valid and reproducible assessment of DILI and HILI cases [15]
● A typical intelligent diagnostic algorithm in line with concepts of artificial intelligence (AI) to solve complex processes by scored items [74]
● A diagnostic algorithm for objective, standardized, and quantitative causality assessment [3,5,13,14,15,16,75]. Summing up the individual scores derived from each key element provides the final causality gradings: score ≤ 0, excluded causality; 1–2, unlikely; 3–5, possible; 6–8, probable; and ≥9, highly probable [15].
● Assessment is user-friendly and cost-effective, with results available in time and without the need for rounds to provide arbitrary opinions [5,7,15]
● Transparency of case data and clear result presentation [5,7,15]
● Suitable for re-evaluation by peers [5] and regional registries, national or international regulatory agencies, and pharma firms [5,15]
● Encourages prospective case data collection to obtain the best results; however, the RUCAM is also prepared for studies with a retrospective study protocol [15]
● Real-time evaluation of the DILI case at the bed side [15]
**Clearly defined and scored key elements [15]**
● Time frame of the latency period
● Time frame of the dechallenge
● Recurrent ALT or ALP increase after drug cessation
● Risk factors
● Individual comedications
● Exclusion of alternative competing causes
● Markers of HAV, HBV, HCV, and HEV
● Markers of CMV, EBV, HSV, and VZV
● Cardiac hepatopathy and other alternative causes
● Liver and biliary tract imaging
● Doppler sonography of liver vessels
● Prior known hepatotoxicity of drug or herb
● Unintentional re-exposure
**Other important specifics [15]**
● Laboratory-based liver injury criteria
● Laboratory-based liver injury pattern
● Liver injury-specific method
● Structured, liver-related method
● Quantitative method, based on scored key elements

Abbreviations: AI, artificial intelligence; ALT, alanine aminotransferase; ALP, alkaline phosphatase; CAM, causality assessment method; CMV, cytomegalovirus; DILI, drug-induced liver injury; EBV, Epstein–Barr virus; HAV, hepatitis A virus; HBV, hepatitis B virus; HCV, hepatitis C virus; HEV, hepatitis E virus; HILI, herb-induced liver injury; HSV, herpes simplex virus; RUCAM, Roussel Uclaf Causality Assessment Method; VZV, varicella zoster virus.

**Table 4:** Challenges and limitations of the RUCAM.

Challenges and Limitations of the RUCAM
● The quality of published RUCAM-based case data strongly depends on the qualification and experience of the submitting physician
● The RUCAM cannot compensate for inadequate-quality data and case providers not familiar with liver diseases; quality problems also remain on the side the reviewers and journal management [76,77,78,79]
● Intentional upgrading of causality levels from possible to probable in cases initially assessed by the objective updated RUCAM and subsequently re-assessed by the global introspection in a report with Western co-authors remains debatable [76], as substantiated in three Letters to the Editor presented by authors from India and Iceland [77], and China [78,79].
● Fraudulent upgrading from possible to probable RUCAM gradings of published cases with the intention to provide more power to risky liver injury, uncovered in court, is outside of any ethical standard [80]
● Challenging are reports titled as DILI but, in fact, several cohorts were lumped together with non-drugs like herbs or so-called dietary supplements as causatives of HILI, providing biased results for drugs and the other causatives due to cohort heterogeneity
● Publications occasionally report on RUCAM-based DILI cohorts that include cases with a possible causality grading, which confounds good data with a probable or highly probable causality level [76]. This problem must be solved prior to submission by deleting all cases with a possible or lower causality grading from the analysis to be published
● Challenging for the RUCAM are mixed cohorts of DILI caused by multiple medicinal products without providing individual RUCAM scores for each product or giving causality gradings as means ± SEM or ± SD for drug groups [3,4]
● Misuses of the RUCAM are reports on DILI without values of the ALT and ALP, preventing both verification of criteria characterizing the liver injury as well as calculation of the R (ratio) and selection of the appropriate RUCAM subtype for correct causality assessment [15]
● Misuses of the RUCAM are attempts including the results of positive unintentional re-exposure without adherence to the specific criteria [15]

Abbreviations: ALP, alkaline phosphatase: ALT, alanine aminotransferase; DILI, drug-induced liver injury; HILI, herb-induced liver injury; RUCAM, Roussel Uclaf Causality Assessment Method.

**Table 5:** Validation of the RUCAM.

Reports on Validation of the RUCAM
● The RUCAM was internally validated using published DILI reports with positive test results for re-exposure, also named positive rechallenge, which demonstrated without incorporation of the rechallenge test into the score a sensitivity of 86%, specificity of 89%, positive predictive value of 93% and negative predictive value of 78% [14]. Such results were commonly appreciated [5] and underlined the value of the original RUCAM as a robust diagnostic algorithm [13]. Positive unintentional re-exposure tests are considered the gold standard among DILI experts [5,14], as erroneous re-exposure of a suspected drug provides in retrospect the strongest evidence of DILI [5] if strict criteria are fulfilled [15]. The good validation data were confirmed by subsequent studies [71,72,73]
● A good reliability based on interrater agreement by using the original RUCAM for DILI cases was reported as a first external study [71]
● A second external study reported that there were no discrepancies in the assessments by the two hepatologists who used the original RUCAM in suspected iDILI cases due to sevoflurane and desflurane [73]. This was a prospective incidence study of 15 patients that provided RUCAM-based causality gradings of highly probable in 3 cases, probable gradings in 5 cases, and possible gradings in 7 patients
● A third external validation study used the updated RUCAM for the determination of causality described in 72 patients with COVID-19 and suspected DILI [72]. Two independent rating pairs (consisting of two clinical pharmacologists plus two general physicians), who had received a short training program for pilot testing just prior to the actual RUCAM use, determined the likelihood of DILI using the RUCAM scale in these DILI patients. As a result, the overall Krippendorf kappa was 0.52, with an intraclass correlation coefficient (ICC) of 0.79, viewed as excellent reliability for using the updated RUCAM [72]. Whether this was achieved through the prior training remains to be verified by assessors without prior training. Confirming previous reports [14,71], this good reliability result was remarkable as based on a retrospective study design [72]

Abbreviations: COVID-19, coronavirus disease 2019; DILI, drug-induced liver injury; RUCAM, Roussel Uclaf Causality Assessment Method.

**Table 6:** Experiences and weaknesses of the US DILI network method [81].

Published Experiences and Weaknesses of the US DILI Network Method
● Cases were enrolled in the registry within 6 months of DILI onset and underwent global introspection syn so called expert opinion
● Causality assessment in real time for clinicians’ use was not feasible
● There was no accepted definition provided for an expert in DILI
● For each case, consensus must be achieved, excluding minority votes
● Consensus is still a subjective opinion
● The network process restricts the naming of offending agents to 3
● Strong opinions or biases of single experts were reported
● Lengthy and lively conversations often occurred during the processes
● The network process is described as cumbersome, time-consuming, and costly, needing data exchanges, monthly meetings, and logistics with administrative, organizational, and technological expertise
● Each case received a final likelihood range as a percentage, arbitrarily assigned by the assessors, not based on individually scored elements
● The total bilirubin was one of the inclusion criteria if >2.5 mg/dL without ruling out unconjugated hyperbilirubinemia due to, e.g., Gilbert syndrome
● Network experts missed the diagnosis of HEV in wrongly diagnosed DILI cases needing a downgrading of the percentage DILI likelihood
● Not using a gold standard, a good method reliability was assumed
● External validation of the method with a different group of experts is explicitly discouraged as labor is considered intensive and expensive
● The network method was only used in US centers
● Despite the weaknesses, the network method is assumed to be best standard for the time being, but it was still imperfect in 2016, asking for mandatory improvements
● Finally, the original RUCAM was surprisingly quoted and described with 11 plain words: “RUCAM requires decline in liver enzymes to get a high score”.

Abbreviations: HEV, hepatitis E virus; DILI, drug-induced liver injury.

**Table 7:** Drugs causing iDILI with a lack of detectable HLA association [81].

Drugs with IDILI and No Detectable Significant Signal in HLA Region
Atorvastatin and other statins
Fasiglifam (TAK-875)
Azathioprine and other thiopurines
Interferon beta
Ciprofloxacin and other fluoroquinolones
Isoniazid
Diclofenac
Nimesulide

Note: Based on >15 cases available for study but no detectable significant signal in the HLA region, compiled from previous data [82].

**Table 8:** Key facts on HLA’s role in iDILI, based on previous publications [83,84,85,86,87,88,89,90].

Role of HLA in the Development of iDILI
● There is a well-documented association of HLA with RUCAM-based iDILI caused by a limited number of drugs
● Drugs implicated in RUCAM-based iDILI are largely metabolized by hepatic microsomal cytochrome P450 isoforms
● In addition, a minority of drugs implicated in RUCAM-based iDILI are metabolized by non-CYPs like alcohol dehydrogenase, aldehyde oxidases, aldehyde dehydrogenase, flavin-containing monooxygenases, and xanthine oxidase
● HLA represents a complex of genes that encode the major histocompatibility complex (MHC) to regulate immunity
● The processes metabolizing the drugs lead not only to harmless metabolites but eventually also to reactive oxygen species (ROS), which in turn trigger the injury of intracellular organelles of the hepatocytes
● The drug or its reactive metabolites function as haptens, bind to proteins, and then form neoantigens that present on specific HLA molecules with the risk of triggering an inappropriate immune response that contributes to the liver injury
● Neoantigens derived from damaged liver cell organelles and toxic drug metabolites attack circulating immune cells, which enter the liver and function there outside of the hepatocytes as resident immune cells and will activate silent immune cells to active immune cells
● The initiation of an immune response requires the activation of antigen-presenting cells (APCs) by molecules such as danger-associated molecular pattern molecules (DAMPs)
● The mechanism by which DAMPs induce an immune response proceeds via the activation of inflammasomes. Although it appears that the liver damage is mediated by the adaptive immune system, an innate immune response is required for an adaptive immune response
● The dominant immune response in the liver is immune tolerance, and it is only when immune tolerance fails that significant liver injury occurs

Abbreviations: DILI, drug-induced liver injury; HLA, human leucocyte antigen; RUCAM, Roussel Uclaf Causality Assessment Method.

## Data Availability

All data are derived from published reports.
